# Nucleotide Sugars in Chemistry and Biology

**DOI:** 10.3390/molecules25235755

**Published:** 2020-12-06

**Authors:** Satu Mikkola

**Affiliations:** Department of Chemistry, University of Turku, 20014 Turku, Finland; satu.mikkola@utu.fi

**Keywords:** nucleotide sugar, glycosylation, glycoconjugate, mechanism, reactivity, synthesis, chemoenzymatic synthesis

## Abstract

Nucleotide sugars have essential roles in every living creature. They are the building blocks of the biosynthesis of carbohydrates and their conjugates. They are involved in processes that are targets for drug development, and their analogs are potential inhibitors of these processes. Drug development requires efficient methods for the synthesis of oligosaccharides and nucleotide sugar building blocks as well as of modified structures as potential inhibitors. It requires also understanding the details of biological and chemical processes as well as the reactivity and reactions under different conditions. This article addresses all these issues by giving a broad overview on nucleotide sugars in biological and chemical reactions. As the background for the topic, glycosylation reactions in mammalian and bacterial cells are briefly discussed. In the following sections, structures and biosynthetic routes for nucleotide sugars, as well as the mechanisms of action of nucleotide sugar-utilizing enzymes, are discussed. Chemical topics include the reactivity and chemical synthesis methods. Finally, the enzymatic in vitro synthesis of nucleotide sugars and the utilization of enzyme cascades in the synthesis of nucleotide sugars and oligosaccharides are briefly discussed.

## 1. Introduction

Nucleotide sugars consist of a monosaccharide and a nucleoside mono- or diphosphate moiety. The term often refers specifically to structures where the nucleotide is attached to the anomeric carbon of the sugar component. Such nucleotide sugars are glycosyl and phosphoglycosyl donors in the biosynthesis of carbohydrates and glycoconjugates in all living organisms [1]. The nucleotide part can also be attached to the non-reducing end of the sugar, as in ADP-ribose. Even though the role of such nucleotide sugars may, to certain extent, be similar, the biological reaction pathways are different [2,3]. Therefore, these “reducing nucleotide sugars” are not discussed in this review.

As glycosyl donors in glycan synthesis, nucleotide sugars take part in processes that are essential for the correct function and survival of living organisms. Glycans are involved in the communication and interactions of cells, and defects in glycosylation may result in serious malfunction, or can even be fatal [4,5,6,7,8,9]. An alteration in glycosylation can indicate a pathological condition, such as cancer [10,11,12,13,14]. Recently, hexosamine pathway producing UDP-*N*-acetylglucosamine (UDP-GlcNAc) has been identified as an important target to influence glycosylation processes [15,16,17].

Hence, enzymes that are involved in the biological processes of nucleotide sugars are potential targets of drug development, and nucleotide sugar analogues are potential inhibitors of these processes [18,19]. As the structure and composition of bacterial carbohydrates differ markedly from those found in the human body, bacterial processes are especially important as targets [20]. Furthermore, foreign or altered glycans activate the defense mechanisms, and oligosaccharide sequences have been studied in vaccines against bacterial [21,22,23,24,25] or fungal [22] infections, as well as cancer [25,26].

Considering these crucial roles of glycans, efficient methods for the synthesis of oligosaccharides are required. Natural and modified nucleotide sugars are needed as starting materials in the synthesis and as inhibitors of biological processes. Efficient synthetic and diagnostic methods are needed, and information on the reactivity and on reaction pathways under biological and chemical conditions support the development of these methods. Various aspects of nucleotide sugars have been studied over the years by scientists working on different fields. Different worlds do not always meet, and this review attempts to bring together the biology and chemistry of nucleotide sugars by concentrating on the molecules and on the details of their reactions.

## 2. Nucleotide Sugars in the Biosynthesis of Glycoconjugates

The idea that sugar nucleotides are sugar donors in the synthesis of oligosaccharides catalyzed by glycosyltransferases is an oversimplification, for the synthesis of even the simplest of glycans is an extremely complex process. While the basics of the carbohydrate synthesis are similar throughout all living species, there are also marked differences. In the following overview, the biosynthesis and roles of the main classes of carbohydrates in human and bacterial cells are briefly described.

### 2.1. Eucaryotic Glycoproteins and Proteoglycans

Proteins and peptides are usually heavily decorated with oligosaccharide sequences and glycans that are attached to amino acid side chains by *O*- or *N*-glycosidic bonds. *N*-linked glycans are usually attached to the γ-amido group of an asparagine moiety. All *N*-linked glycans share the same branched core sequence that consists of two GlcNAc units and three mannoses, but they differ in the number and composition of the side chains [27]. *N*-linked glycans serve in an important role in the quality control in the glycoprotein synthesis [7,28,29]. *N*-glycans are also involved in interactions of cell-surface ligands and receptors, and it has been shown that a mutation of certain glycosyltransferases involved in *N*-glycosylation can result in serious birth defects [7]. Altered *N*-glycosylation has been identified also as a biomarker for diabetes [17].

*O*-linked glycans are attached to an HO group of a serine or threonine residue. Their structures are more variable than those of *N*-linked glycans [30]. The best-known class of *O-*glycosylated proteins is mucins, which are heavily glycosylated and sulfated proteins of high molecular weight [10,12]. The roles of mucins are diverse. Secreted mucins are typically lubricants or serve as protection against pathogens, whereas membrane-bound mucins are involved in signaling pathways [10,11,12,13]. Altered glycosylation patterns are observed in various diseases, and such modifications are particularly well-characterized in several types of cancer [10,11,12,13].

*O*-mannosylation is another common type of protein *O*-glycosylation [9,30,31]. *O*-mannosyl glycans are important for brain and muscle development, and defects in *O*-mannosylation have been identified as the primary cause for some forms of muscular dystrophies [9].

Glycosaminoglycans (GAG) form a different class of *O*-linked glycans. Glycosaminoglycans are linked to a serine or threonine by a tetrameric linkage region that is common to all GAG molecules. The GAG sequence itself is linear and constitutes of disaccharides that contain one hexuronic acid or galactose and a hexosamine [8,32]. Apart from hyaluronan, GAGs are sulfated at various positions and attached to a peptide to form proteoglycans [33]. Glycosaminoglycans are involved in various physiological and pathological processes through their interactions [32,33,34,35]. These interactions have been very recently collected in a database listing 932 GAG–protein interactions [36]. GAGs have been associated with various diseases, including cancer [14,34,35] and Alzheimer’s disease [34]. Defective GAG synthesis is also the primary cause of several genetic disorders affecting bones and connective tissues [8].

The biosynthesis of *N*-linked glycans [7,27] and *O*-mannosyl glycans [5,6] occurs in the endoplastic reticulum (ER). Membrane-bound dolichol phosphates (Dol-P) serve as anchors that attach the starting materials and the growing oligosaccharides to the membrane [6]. The first steps of the synthesis occur on the cytosolic side of the membrane, where the reaction of Dol-P with nucleotide sugars produces dolichol-phosphate-mannose (DPM) and dolichol-phosphate-glucose (DPG) as well as dolichol diphosphate *N*-acetyl glucosamine (Dol-P-P-GlcNAc) [6]. Mechanisms of these processes are discussed in more detail in Section 4.1 and Section 4.2.

The core sequence of *N-*linked glycans is synthesized on a Dol-P-P-GlcNAc anchor using sugar nucleotides as sugar donors [7,27]. The synthesis continues in the lumen of ER after the precursor molecule has been flipped through the membrane. DPM and DPG serve as glycosyl donors in the lumen. Once the precursor is complete, it is transferred from the dolichol pyrophosphate to the γ-amido group of an asparagine moiety by an oligosaccharyltransferase enzyme, and the resulting glycoconjugate is transported to Golgi apparatus for further trimming and quality control [6].

*O*-mannosyl glycan synthesis begins in the lumen of ER, where DPM acts as the mannosyl donor [5,6]. The mannosylated peptide is transported to the Golgi, where the glycan chain is elongated by further glycosyl transfers from sugar nucleotide donors. The synthesis of mucin-type glycans occurs also in the Golgi, where it is initiated by an UDP-GalNAc:polypeptide *N-*acetylgalactosinylstransferase that transfers a sugar from UDP-*N*-acetylgalactosamine (UDP-GalNAc) to a serine or threonine [10,11,12,13]. Further glycosylations are catalyzed by specific membrane-bound glycosyl transferases that locate in different compartments of the Golgi and form assembly lines for glycoprotein production [10,13].

GAGs are also synthesized in the Golgi, where the process is initiated by a transfer of xylose from UDP-xylose to a serine residue by a β-xylosyltransferase [8,32,37]. The synthesis of the linkage region is completed by the transfer of two galactoses and a glucuronic acid from their respective UDP sugars. The second stage of the process is the synthesis of the GAG polymer sequence by consecutive glycosyl transfer reactions. At the final stage, the polymer is sulfated.

### 2.2. Bacterial Cell-Wall Carbohydrates

Bacteria have developed different types of cell envelope architectures for protection. The cell envelope of Gram-positive bacteria consists of one lipid bilayer and a thick peptidoglycan layer, whereas in Gram-negative bacteria, there are two lipid bilayers and a thinner peptidoglycan in between them [38,39]. The carbohydrate component of peptidoglycan is an oligosaccharide consisting of alternating *N*-acetylglucosamine (GlcNAc) and *N*-acetylmuramic acid (MurNAc) residues, which are both derived from UDP-GlcNAc [40,41]. Oligosaccharides are crosslinked by oligopeptides attached to MurNAc residues, and a mesh-like structure is formed. The cell-surface is decorated with glycoconjugates, such as wall teichoic acids and lipoteichoic acids in Gram-positive bacteria [20,42,43,44] and lipopolysaccharides in Gram-negative bacteria [39,45,46]. Both Gram-negative and Gram-positive bacteria can be further protected by a capsule that consists of oligosaccharides [21,23,43,47,48,49,50]. In the following four different types of carbohydrate structures, capsular polysaccharides, *O*-antigen carbohydrates, wall teichoic acids, and lipoteichoic acids are briefly discussed.

#### 2.2.1. Capsular Polysaccharides

Capsular polysaccharides (CPS) typically consist of short repeating units that may be linked by phosphodiester bonds [51,52]. The polysaccharide can be a homopolymer or consist of short repeating oligosaccharide sequences [21,49,50,51]. They can be branched [47] or partially acetylated at various positions [21,49]. Same species may express different polysaccharides [23,49,52], which may be synthesized by different mechanisms [48].

CPS polymerization takes place on the cytosolic side of the inner lipid membrane, and three main pathways have been identified: the Wzy pathway [23,47,52,53,54], synthase-dependent pathway [47,54], and ABC transporter-dependent pathway [21,48,53,54]. In the Wzy-dependent process, the polymer is constructed of short repeat units. They are synthesized on polyprenyl phosphate by consecutive transfers of sugars or phosphosugars from corresponding nucleotide sugars [23,47,51,52,53,54]. A complete repeat unit is flipped across the membrane into the outer side by Wzy flippase, where a Wzy polymerase attaches it to a growing polysaccharide. Then, the polymer is transferred to peptidoglycan or a membrane component. Alternatively, it may also be released as an exopolysaccharide.

The ABC-dependent pathway is different in that the carbohydrate polymer synthesis is processive, and the polymer is transported through the membrane by an ATP-dependent ABC transporter [48,53]. In the synthase-dependent pathway, the growing oligosaccharide chain is attached to a membrane-bound phosphatidyl glycerol, and only one transferase is involved in the synthesis [47].

#### 2.2.2. Lipopolysaccharides

Lipopolysaccharides (LPS) typically consist of a lipid anchor (Lipid A) that links the glycoconjugate to the outer cell membrane, a core polysaccharide and, in most cases, a repeat-unit polysaccharide called o-antigen (O-PS) [55,56]. The inner core is well conserved within the same species, but structures from even distantly related species can share similar features. Heptose sugars and 3-deoxy-d-manno-oct-2-ulosonic acid (Kdo) are typical constituents of the inner core. The outer core is structurally more diverse even within the species of the same family.

An O-antigen oligosaccharide is linked by a glycosidic bond to a monosaccharide unit of the outer core [55]. The repeat-unit oligosaccharides are structurally very diverse [55], and a large number of different monosaccharides have been identified [57]. Acetyl, phosphoglyceryl, or glycosyl side chains bring about further variation [58,59]. The O-antigen backbone may also contain glycerol or ribitol units and/or phosphodiester bonds [60]. The carbohydrates are synthesized on undecaprenyl pyrophosphate (Und-PP)-linker through the Wzy or ABC transporter-dependent pathway [46].

#### 2.2.3. Teichoic Acids

Teichoic acids (TA) are cell membrane-bound carbohydrate polymers found in Gram-positive bacteria that generally consist of repeating glycerolphosphate or ribitolphosphate-containing units [42,43,44,45,61,62,63]. The backbone can be further modified by saccharyl, phosphocholinyl, or amino acid substituents [20,42,64,65]. Teichoic acids are classified as lipoteichoic acids (LTA) or wall teichoic acids (WTA) based on the membrane attachment. LTA are attached to the membrane by a lipid anchor [42,44], whereas WTA are attached to peptidoglycan by a highly conserved disaccharide linkage unit [20,43]. Five different WTA types [20] and four different LTA types [42,44] of increasing complexity have been identified.

The teichoic acid synthesis occurs in the cytosol on the membrane-bound undecaprenyl phosphate (Und-P) [20,42,44,63]. The first steps in the WTA synthesis are conserved: corresponding transferases transfer GlcNAc and *N*-acetyl mannosamine (ManAc) from their respective uridine diphosphate (UDP) sugar donors, and glycerol phosphate is transferred from cytidine diphosphate glycerol (CDP-glycerol) by a glycerophosphotransferase. The polymer chain is generally synthesized first, and the side chain glycosyls are added at the last step. Once the polymer is complete, it is flipped through the membrane and attached to peptidoglycan via a phosphodiester linkage to C6 of a MurNAc residue [20].

The synthetic pathways for LTA are more diverse [42,44]. For example, in *S. pneumoniae,* LTA and WTA are structurally similar, and they are produced by the same machinery [42]. In the case of LTA type I from *S. aureus*, the glycerol phosphate polymer is built by transferring glycerol phosphates from the membrane-bound phosphatidyl glycerol donor [42,44].

## 3. Structure and Biosynthetic Routes of Nucleotide Sugars

While nucleotide sugars serve as glycosyl donors in species of all living organisms, and the basic pathways are similar, the variety of structures is different. In the following, the structures and biosynthetic routes of nucleotide sugars found in mammalian, plant, and bacterial cells are discussed.

### 3.1. Structures and Biosynthetic Routes of Mammalian Nucleotide Sugars

**Figure 1:**
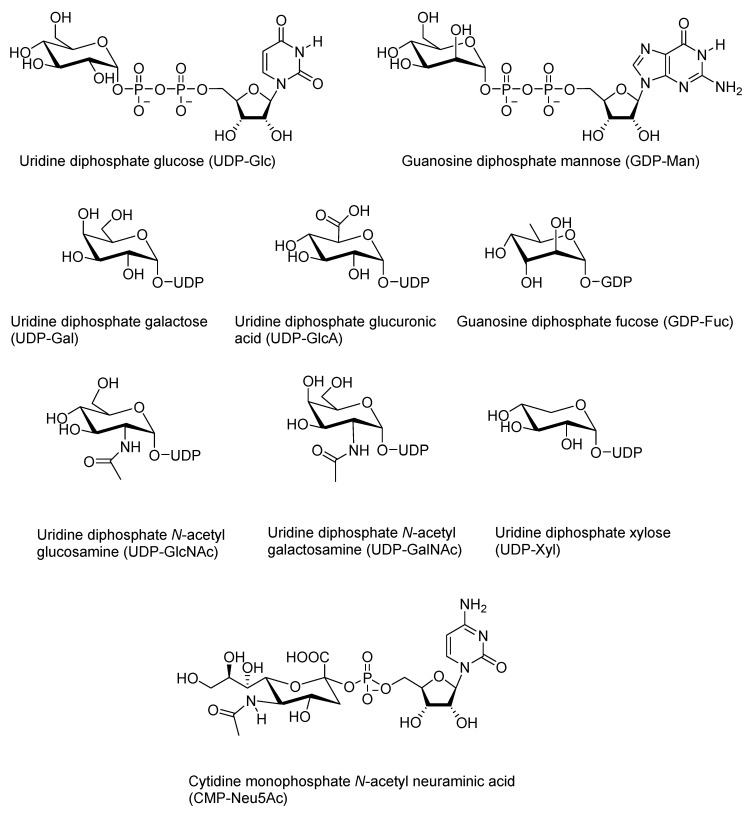
Structures of common nucleotide sugars found in human cells.

The number of different monosaccharides, and hence, also of nucleotide sugars, in mammals is fairly limited. Out of the nine most abundant ones in human cells, glucose (Glc), galactose (Gal), *N*-acetyl glucose (GlcNAc), *N*-acetyl galactose (GalNAc), glucuronic acid (GlcA), and xylose (Xyl) are activated as UDP sugars, and mannose (Man) and fucose (Fuc) are activated as GDP derivatives [66]. Sugars are in d-configuration, apart from fucose, which is an l-sugar. Neuraminic acid (NeuAc) is different in that it is activated as a cytidine monophosphate. Another difference is the site of production: while other sugar nucleotides are synthesized in the cytosol, human CMP-NeuAc is synthesized in the nucleus [4,67]. The structures of the common nucleotide sugars in human cells are shown in Figure 1.

There are two main biosynthetic pathways for nucleotide sugars [4,66,68,69]: de novo synthesis using dietary monosaccharides as the starting material and salvage pathways that recycle monosaccharides. The pathways are interlinked, and the same nucleotide sugar can be synthesized through different pathways. Of the dietary sources, glucose and fructose sugars are the most important ones. After they have been taken up by a cell, they are phosphorylated to sugar 6-phosphates. Glucose 6-phosphate is converted to glucose 1-phosphate that, in a reaction with uridine triphosphate (UTP), produces UDP-glucose (UDP-Glc). The formation of UDP-Glc is reversible, and it is catalyzed by glucose 1-uridyltransferase or UDP-glucose pyrophosphorylase [70]. UDP-Glc can be oxidized to UDP-glucuronic acid by UDP-glucose dehydrogenase. Consecutive decarboxylation produces UDP-xylose [71]. UDP-galactose (UDP-Gal) is formed in an exchange reaction of galactose-derived galactose 1-phosphate and UDP-Glc catalyzed by galactose 1-phosphate uridylyltransferase [70].

Fructose 6-phosphate is a precursor for the biosynthesis of *N*-acetylglucosamine and mannose 1-phosphates, which are converted to corresponding UDP and GDP sugars, respectively [4]. UDP-GlcNAc can be further isomerized to UDP-GalNAc. In turn, GDP-mannose, can be converted to GDP-L-fucose. UDP-GlcNAc is a starting point of the synthesis of CMP-NeuAc: UDP-GlcNAc is converted in three steps through mannose 6-phosphate to neuraminic acid, which reacts with cytidine triphosphate (CTP) to produce the nucleotide sugar [64]. GDP-fucose, GDP-mannose, UDP-GalNAc, and UDP-GlcNAc are formed also through salvage pathways from recycled monosaccharides and ATP [68].

Nucleotide sugar composition varies in different species, even though there are also striking similarities. The variety in such distant eukaryotic species as *Plasmodium falciparum*, a unicellular parasite [72], or a nematode *Caenorhabditis elegans* [73] is surprisingly similar to that in human cells. The biosynthetic routes for the most common nucleotide sugars are also similar [72]. An interesting observation is that UDP-galactofuranose and TDP-rhamnose that are typically found in prokaryotes were identified in *C. elegans*, but glycoconjugates containing the corresponding sugars were not found [73].

### 3.2. Nucleotide Sugars in Plants

A more significant difference is observed between plant and animal kingdoms: some thirty different nucleotide sugars have been identified in plants [74]. The basic pathways utilizing fructose or glucose are the same as described above, but the branches are extended [74,75,76,77]. Thus, in plant cells, for example, UDP-GlcA gives rise to the formation of a branched sugar apiose (Api) in the form of UDP-Api together with UDP-Xyl. UDP-Xyl is further epimerized to UDP-l-arabinopyranose (UDP-l-Ara*p*), which is interconverted with its furanose isomer UDP-l-Ara*f* [74,77].

Sialic acids are not found in plant carbohydrates, but carboxylic acid containing 3-deoxy-d-manno-oct-2-ulosonic acid (Kdo) is a constituent in rhamnogalacturonan II pectins along with other acidic or rare monosaccharides [78]. Similarly to bacterial Kdo and human sialic acids, it is activated as a CMP sugar [74,76,78]. Aceric acid is another carboxylic acid function containing monosaccharide in plants [78], but its activated form is not known.

In addition to the wider variety of monosaccharides, plant cells contain nucleotide sugars with nucleotide–sugar combinations not typically found in human carbohydrates. Some of them are common, such as ADP- and GDP-α-d-glucose, which serve in important roles as the precursors for the synthesis of starch and glucomannan, respectively [74]. l-Galactose (l-Gal) sugar found in xyloglucans [79] is activated as GDP-l-Gal [76,78], which is another example of an unusual combination. Several rare nucleotide sugars, including thymidine diphosphate (TDP) derivatives, or enzymes pointing at them, have been identified in plants, but the roles of these nucleotide sugars are not known [74,78].

### 3.3. Bacterial Nucleotide Sugars

The number of different monosaccharide units in prokaryotes is even larger [74]. The structural variety is particularly remarkable in O-antigen polysaccharides: more than 60 monosaccharides and 30 non-carbohydrate units have been identified [44]. A large diversity has been observed also in capsular polysaccharides. Results on extensive studies on *Acinetobacter baumanii* have been collected in a database of gene clusters involved in the biosynthesis of CPS and outer core polysaccharides [80]. Genes encoding enzymes involved in the synthesis of 24 different nucleotide sugars have been identified thus far in a single bacterial species. Some of the nucleotide sugars are rare and found only in certain strains, such as some CMP-ulosonic acid derivatives [81].

Samuel and Reeves [57] have described the biosynthetic routes for 30 O-antigen nucleotide sugars in a review that is organized based on the sugar nucleotide pathways. It is easy to see that the activating nucleotide for a given sugar is conserved in several kingdoms of life. Thus, for example, the activated form of l-fucose is GDP-l-fucose in human [4], plant [74], and bacterial [57] carbohydrates. Similarly, human [67] and bacterial sialic acids [57], as well as Kdo in plants [74,78,82] and bacteria [44,82,83], are activated as CMP sugars. The biosynthetic pathway for Kdo has been reported to be almost completely conserved between plants and bacteria [82].

Bacteria use a wider variety of nucleotides in the activation of sugars than eukaryotes do. TDP sugars are common, and some bacteria also use CDP sugars in carbohydrate synthesis [57]. TDP and CDP-activated sugars are usually 6-deoxy sugars, as in TDP-l-rhamnose or TDP-d-fucose, or 3,6-dideoxy sugars as in CDP-paratose (3,6-dideoxy-d-*ribo*-hexose) or CDP-tyvelose (3,6-dideoxy-l-*arabino*-hexose). The alditol units ribitol and glycerol are found in many capsular polysaccharides and are also activated as CDP derivatives [20]. CDP-glycerol is synthesized from glycerol 3-phosphate and CTP [84]. Ribulose 5-phosphate is the starting material in the synthesis of CDP-ribitol [85].

The heptose sugars found in the inner core of lipid A are derived from ADP-l-*glycero*-d-*manno*-heptose and GDP-d-*glycero*-d-*manno*-heptose [44,57]. Sedoheptulose 7-phosphate is the starting material in their biosynthesis. These syntheses are exceptional in that the nucleotide is added to the sugar at a late stage of the oligosaccharide synthesis. Generally, sugar modifications are carried out at the nucleotide sugar level, as for example in the synthesis of another inner core carbohydrate, 4-amino-4-deoxy-l-arabinose. The corresponding nucleotide sugar is synthesized from UDP-glucose in the cytoplasm [86].

## 4. Mechanisms of Enzymatic Reactions of Nucleotide Sugars

As has been shown in previous chapters, the synthesis of nucleotide sugars and carbohydrates involves a vast number of enzymatic processes that build, modify, and cleave nucleotide sugars. This chapter discusses the mechanisms of reactions that result in the decomposition of nucleotide sugars, i.e., glycosylation, phosphoglycosylation, and hydrolysis. These reactions are depicted in the following schemes.

### 4.1. Catalysis by Glycosyl Transferases

**Scheme 1:**
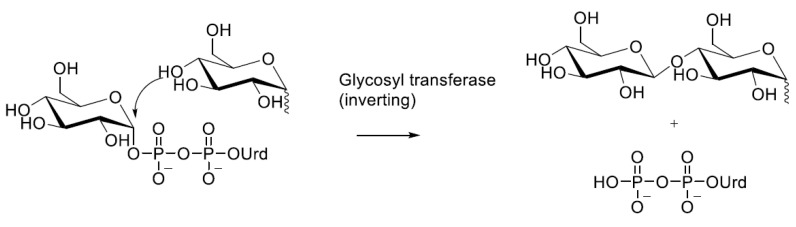
The reaction catalyzed by glycosyl transferases: A nucleophilic attack by an HO group at the reducing end of the acceptor on the anomeric carbon of a nucleotide sugar donor; a glycosidic bond is formed.

Glycosidic linkages of oligosaccharides and glycans are formed in glycosylation catalyzed by glycosyl transferases that transfer a glycosyl moiety from a glycosyl donor to an acceptor [1,87]. Leloir transferases use nucleotide sugars as donors, and a nucleoside diphosphate or monophosphate is released as the leaving group in the reaction (Scheme 1). The glycosyl acceptor in the glycan synthesis can be another sugar molecule, or it can be an amino or hydroxy group of a peptide side chain. [88,89]. Glycosyl transferases may glycosylate also other acceptors, such as modified nucleic acid bases [90].

Glycosyl transferases (GT) are classified on the basis of their structure and the stereochemical outcome of the glycosylation reaction. Based on the protein fold, they are divided into five categories [87,91]. Enzymes belonging to GT-A, GT-B, GT-D, and GT-E superfamilies are Leloir transferases using nucleotide sugars as sugar donors [91]. Most of the glycosyl transferases fall in well-known GT-A or GT-B categories, whereas GT-D [92] and GT-E [93] superfamilies have been recently discovered, and only a few enzymes have been identified. Enzymes are divided further into families on the basis of amino acid sequences, and as a consequence, members of the same family may well differ in their donor or acceptor specificity [1].

Glycosyltransferases are either retaining or inverting based on the stereochemistry of the glycosylation reaction, and the stereochemistry is independent of the protein fold. Inverting glycosyltransferases catalyze an S_N_2-type direct displacement reaction at the anomeric carbon of the sugar donor [1,88,94]. The nucleophilic group is activated by general base catalysis. Basic amino acid side chains such as a carboxylate or an imidazole group usually act as bases. GT-A transferases typically contain a well-conserved metal ion-binding motif that is not found in GT-B enzymes. The coordinated metal ions, Mn^2+^ or Mg^2+^, assist the departure of the leaving nucleoside diphosphate by stabilizing the forming negative charge. However, also, metal ion-independent GT-A transferases are known. Metal ion-independent glycosyl transferases of either GT-A or GT-B fold stabilize the leaving group by interactions to amino acid side chains through ionic or hydrogen-bonded interactions [95]. Metal ions are still needed for the full catalytic activity of GT-B transferases, but the role of metal ion cofactors appears to be different. A secondary role in product release has been proposed in the catalysis by a DNA-glycosylating β-glucosyltransferase [90].

TagA, the founding member a newly discovered GT-E fold that catalyzes the transfer of the GlcNAc moiety in the synthesis of the disaccharide linkage unit of WTA, is an inverting glycosyl transferase [93]. The S_N_2-type reaction proceeding through an oxocarbenium-like intermediate is independent of metal ions, and the developing negative charge on the leaving group is stabilized by an interaction with an arginine moiety [93].

In contrast to a well-established mechanism for inverting glycosyl transferases, the mechanism of reverting enzymes has been under debate [1,88,94,96]. The debate is focused on two alternatives: a traditional double-displacement with a covalent enzyme–substrate intermediate, and a more unconventional front-side S_N_*i*-like substitution with an oxocarbenium ion-like ion pair intermediate [1,88]. The former mechanism is analogous to the mechanism utilized by retaining glycosyl hydrolases, and it has been supported by chemical rescue experiments using azide ions as external nucleophiles [97]. Steric constraints that would hinder the S_N_*i*-like substitution by a retaining Kdo transferase WbbB2–401 argue also against the S_N_*i*-like mechanism [98]. On the other hand, an X-ray crystallographic study with an unreactive UDP-Glc analogue and α-1,4-galactosyltransferase C (LgtC) from *Neisseria meningitidis* failed to reveal a suitably positioned nucleophilic enzyme side chain [99]. A similar observation was made with a crystal structure of a ternary donor-acceptor-Mn^2+^ complex within the glucosyl-3-phosphoglycerate synthase GpgS from *Mycobacterium tuberculosis* [100].

A number of QM/MM studies on different enzyme systems have also been reported over the years [96,100,101,102,103,104]. While all these studies support the S_N_*i*-like mechanism as the predominant reaction pathway, some studies have also revealed amino acid side chains that could be catalytically significant [96,98,102,103]. Such observations have been proposed to show that a nucleophile can assist the reaction by non-covalent interactions with the oxocarbenium ion [102]. Hence, nucleophilic catalysis can be seen as the extreme of oxocarbenium ion stabilization, and the mechanisms can be seen as a continuum from one extreme to another depending on the possible substrate–enzyme interactions [96,100,101]. It is also generally accepted that glycosyl transferases of family 6 catalyze a glycosylation by the double-displacement mechanism [88,96,101].

### 4.2. Catalysis by Polyisoprenol-Phosphate Glycosyltransferases

**Scheme 2:**
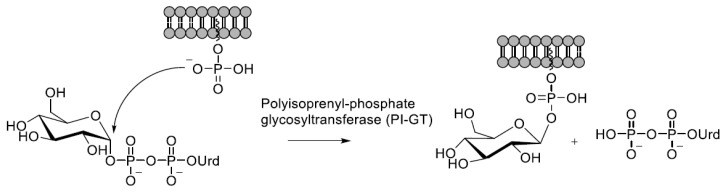
Reaction catalyzed by polyisoprenyl-phosphate glycosyltransferases: A membrane bound polyprenyl phosphate acceptor attacks on the anomeric carbon of the glycosyl donor; a phosphodiester bond is formed.

Polyisoprenyl-phosphate glycosyltransferases (PI-GT) are involved in the synthesis of the membrane-bound Dol-P-Man or Dol-P-Glc glycosyl donors or of their prokaryotic polyprenyl counterparts [6]. The lipid-bound phosphate attacks on the anomeric carbon and a nucleoside diphosphate is released as the leaving group (Scheme 2).

The mechanistic information available is based on crystallographic studies with glucose-specific [105] and mannose-specific [106] enzymes from prokaryotic sources. In both structures, a single metal ion (Mn^2+^ or Mg^2+^) that is coordinated to two carboxylic acid side chains forms a chelate binding to both phosphates in the pyrophosphate. According to the mechanism proposed by Gandini et al. [106], the attacking phosphate is positioned by interactions to arginine and serine side chains, whereas in the mechanism reported by Ardiccioni et al. [105], an aspartic acid acts as a general acid that protonates the leaving UDP.

Both enzymes catalyze the reaction with inversion of the anomeric configuration. Similarities between enzymes from different sources have been taken as an indication of a similar reaction mechanism for all PI-GTs [105,106]. However, it has been pointed out that there are PI-GTs that catalyze the glycosyl transfer with retention of configuration, and a mechanism with an oxocarbenium ion-like transition state has been proposed [107].

### 4.3. Catalysis Phosphoglycosyl Transferases

**Scheme 3:**
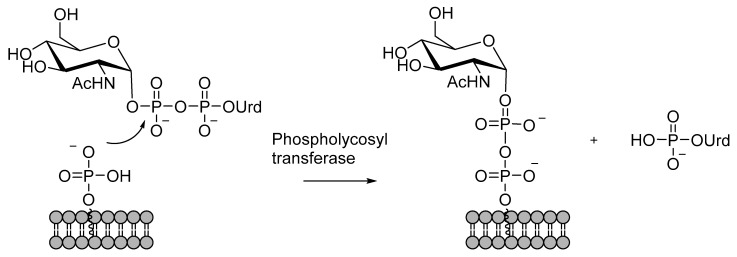
The reaction catalyzed by phosphoglycosyl transferases: a membrane-bound polyprenyl phosphate acceptor attacks the β-phosphate; a pyrophosphate linkage is formed.

Phosphoglycosyl transferases (PGT) are enzymes that transfer a phosphoglycosyl moiety to Dol-P or Pren-P, forming a pyrophosphate bridge that binds a glycosyl moiety or a glycan to the cell membrane (Scheme 3) [108,109]. A nucleoside monophosphate is released as the leaving group. Phosphoglycosyl transferases are divided into two superfamilies that differ in enzyme topology and mechanism of action [109]. One of the superfamilies is characterized by a polytopic membrane architecture. A mechanistically well-known example of this superfamily is the phosphoglycosyl transferases of the WecA family that transfer GlcNAc 1-phosphate from UDP-GlcNAc to polyprenylphosphate or dolicholphosphate in bacteria or eukaryotes, respectively. A thorough kinetic analysis has shown that the catalytic mechanism is a one-step reaction involving a ternary complex of the donor, the acceptor, and an Mg^2+^ ion [110]. The catalytic Mg^2+^ ion is bound to both phosphates in the pyrophosphate moiety, thus enhancing the electrophilicity. The lipid-bound phosphate attacks the β-phosphate, and the attack is assisted by general base catalysis by an aspartate residue that has been shown to be essential by mutating it into alanine [110,111].

Phosphoglycosyl transferase PglC from *Campylobacter consicus* is an example of the other family, dual domain phosphoglycosyl transferases. Mechanistic studies on the enzyme have shown that the reaction is a two-step process with a covalent intermediate [112,113]. A nucleophilic side chain attacks on the phosphate, forming an intermediate that is attacked by the prenyl phosphate in the second step. An Mg^2+^ ion has been shown to be essential for the catalysis, and it is believed to activate the phosphate toward the nucleophilic attack. An aspartate in a conserved Asp–Glu dyad has been proposed as the attacking nucleophile, while the glutamic acid residue has been speculated to assist the release of UMP and nucleophilic attack by Pren-P.

The key observation in the mechanistic studies referred to above is the difference in timing of the UMP release. Imperiali and her group [112] developed a luminescence-based method to follow the release of UMP and showed that in the reaction catalyzed by PglC, UDP-bacillosamine (UDP-diBacNAc) releases UMP in the absence of the Pren-P acceptor, which is consistent with the formation of a covalent intermediate. Al-Dabbagh et al. [110] used radiolabeled UMP* and showed that an exchange reaction between UMP* and UMP from UDP-diBacNAc takes place only in the presence of the acceptor. This shows that the nucleophilic attack and the release of the leaving group are concerted, and that the mechanisms studied by the two research groups are different.

### 4.4. Catalysis by Enzymes Involved in the Formation of Phosphodiester-Linked Carbohydrates

**Scheme 4:**
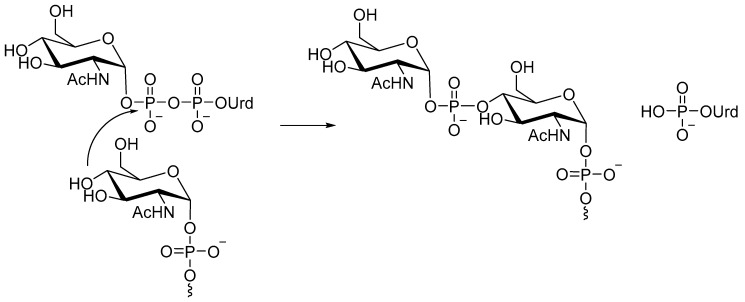
The reaction catalyzed by hexose phosphotransferases: a nucleophilic attack by an HO group at the reducing end of the acceptor on the β-phosphate; a phosphodiester bond is formed.

Enzymes belonging to the Stealth enzyme family catalyze the transfer of a hexose 1-phosphate to a sugar acceptor (Scheme 4) [114]. In the reaction, the acceptor hydroxy group attacks the phosphate in the nucleotide sugar donor, and a phosphodiester bond is formed [115,116,117,118]. In contrast to the enzymatic mechanisms described above, fairly little is known about the mechanistic details of these reactions.

Enzymes from different sources have different metal ion preferences. While catalysis by *N*-acetylglucosamine 1-phosphotransferase from *N. meningitidis* is enhanced by the addition of Mg^2+^ [117], a mannosyl-1-phosphotransferase from *C. difficile* exhibited the highest activity in the absence of metal ions, and the effect of Mg^2+^ was even slightly inhibitive [118]. A eukaryotic xylophosphoryl transferase from *Cryptococcus neoforms* has been reported to be dependent on Mn^2+^ ions [115].

Polyglycerol and polyribitol sequences in WTA are synthesized by a group of Tag and Tar enzymes [20,119]. TagF and TarL are polymerases that synthesize poly(glycerol-3-phosphate) and poly(ribitol-5-phosphate) chains on the disaccharide linkage region using CDP-glycerol and CDP-ribitol donors, respectively. TagF has been co-crystallized with CDP-3-glycerol [120], and an S_N_2 mechanism at the phosphate catalyzed by two histidine residues has been proposed. Mutation studies revealing two essential histidine residues [121] and a thorough kinetic analysis [122] support this mechanism.

TagF-like features have also been observed with Cs1b, which is a capsule polymerase from *N. meningitidis* [123]. This enzyme turned out to be responsible for the transfer of both glycosyl and glycosyl phosphate units exhibiting both glycosyl and hexose-1-phosphate transferase activities [123]. A structural analysis identified two separate domains, one of GT-A fold, and a GT-B folded domain similar to that of TagF. Similarly to TagF, two essential histidine residues have been identified, and the same role in catalysis has been suggested. More recently, a whole family of multidomain polymerases catalyzing the synthesis of capsular polysaccharides has been identified [124].

### 4.5. Hydrolysis of Nucleotide Sugars

**Scheme 5:**
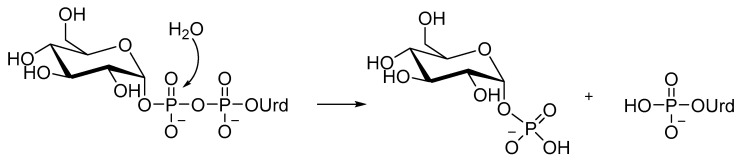
The reaction catalyzed by pyrophosphorylases or hydrolases: a water molecule attacks the phosphate, and the pyrophosphate linkage is cleaved to two monophosphates.

Hydrolysis of the pyrophosphate bridge (Scheme 5) is catalyzed by two types of enzymes: nucleotide sugar pyrophosphorylases that are involved in a reversible nucleotide sugar producing/hydrolysing process that controls the sugar metabolism [69,70], and by NUDIX hydrolases that serve in housecleaning roles and hydrolyze various nucleoside pyrophosphate derivatives [125,126].

As mentioned in Section 3, UDP-Glc is produced by a UDPGlc pyrophosphorylase catalyzed reaction between Glc-1-phosphate and UTP. The reaction is reversible, and the same enzyme catalyzes also the hydrolysis UDP-Glc to UMP and glucose-1-phosphate [69]. The direction of the reaction is dependent on the concentration of the reaction components that affects the polymerization/depolymerization of the enzyme [127]. While the enzyme is found in mammalian, plant, and bacterial cells, the degree of polymerization may be different [127,128,129]. The catalysis by nucleotide sugar-producing pyrophosphorylases has been extensively studied, but these studies usually concentrate on the forward reaction: the formation of UDP-Glc [129,130]. Less is known about the mechanistic details of the reverse hydrolysis reaction. Catalysis is known to be dependent on Mg^2+^ ions [129], and a single Mg^2+^ chelated to two phosphates is observed in X-ray structures [131,132].

NUDIX hydrolases are a family of enzymes that are characterized by a NUDIX box, a conserved sequence of 23 amino acids, and the ability to cleave nucleotide pyrophosphate derivatives. A single enzyme may be able to hydrolyze different types of compounds, but not all of them accept nucleotide sugars as substrates [126]. NUDIX hydrolases generally cleave a nucleotide sugar producing a nucleoside monophosphate and a sugar phosphate as products [125,133]. However, GDP-mannose mannosyl hydrolases (GDPMH) are an exception, and mannose and guanosine diphosphate are formed [134,135]. Nucleotide sugar hydrolyzing NUDIX hydrolases are Mg^2+^-dependent enzymes [136,137,138,139], and one Mg^2+^ ion is required in the catalysis [133,134]. Zn^2+^, Mn^2+^, Ca^2+^, and Cu^2+^ exhibit a 20–30% activity in comparison to Mg^2+^ [138]. The optimal pH for the catalysis by NUDIX hydrolases is high: values between pH 9.0 and 9.5 have been reported [134,139].

Two glutamates [136,137] and one histidine [133] have been observed to be essential for the catalysis. A histidine moiety has been identified as a general base that deprotonates the attacking water molecule [133], while the role of the glutamates is to coordinate the Mg^2+^ ion that enhances the electrophilicity of the phosphate [136]. In contrast to the associative mechanism generally observed with NUDIX hydrolases, GDPMH catalyze the reaction by a dissociative mechanism [135]. Multiple interactions to the leaving diphosphate have been taken as an indication of the importance of the leaving group stabilization: in addition to a Mg^2+^ ion chelating the phosphates, interactions with four amino acid residues have been observed in an X-ray structure [135].

A clearly different reaction mechanism has been shown for a non-NUDIX pyrophosphorylase LpxH involved in the synthesis of lipid A [140,141]. This enzyme is dependent on Mn^2+^, and a metal ion-dimer is involved in the catalysis: one of the Mn^2+^ ions is coordinated to the α-phosphate, while a hydroxo ligand attached to the other one attacks the α-phosphate. As a result, the pyrophosphate bridge is cleaved, and a uridine monophosphate is released [140,141]. A corresponding reaction in species lacking the LpxH enzyme is catalyzed by a Mg^2+^-dependent enzyme that enhances a nucleophilic attack on the β-phosphate [142].

## 5. Chemical Reactions of Nucleotide Sugars

**Scheme 6:**
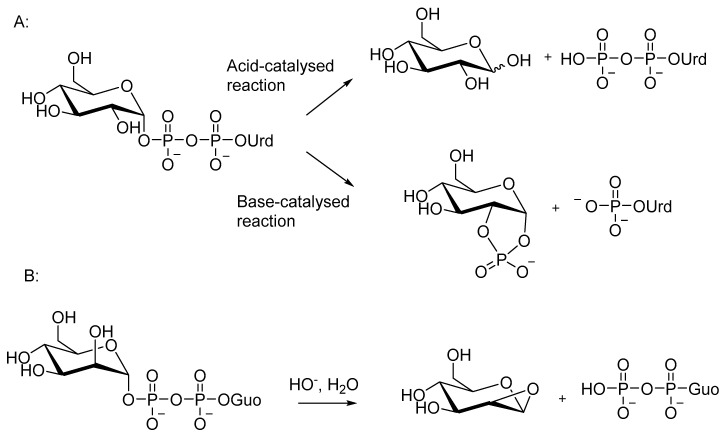
Chemical decomposition of nucleotide sugars in aqueous solutions. (**A**): Acid and base-catalyzed reactions of a nucleotide sugar with a neighboring HO-group in *cis*-orientation. (**B**): Base-catalyzed reaction of GDP-mannose.

As was discussed in Section 4, nucleotide sugars serve in biological processes as glycosyl or phosphoglycosyl donors, and enzymes enhance reactions where an intermolecular nucleophile attacks on the anomeric carbon or on the phosphate. In the absence of enzymes, nucleotide sugars undergo a substitution at the anomeric carbon only under acidic conditions, where the oxocarbenium ion formed upon departure of the diphosphate moiety is stable enough to exist as an intermediate [143]. Under neutral and alkaline conditions, nucleotide sugars react by an intramolecular substitution at the phosphate, and two monophosphates are formed as products [143,144,145,146,147,148]. The acid and base-catalyzed reactions of UDP-Glc are shown in Scheme 6A. The difference in the reactivity is probably one of the reasons why nucleotide sugars are usually not used as glycosyl donors in the chemical synthesis of oligosaccharides.

Under neutral and alkaline conditions, a neighboring HO group of the sugar attacks the phosphate, and a nucleoside monophosphate and a 1,2-cyclic sugar phosphate are formed (Scheme 6A) [143,144,145,146,147,148]. A *cis* orientation of the nucleophile and the phosphate is required for an efficient reaction; GDP-mannose with the adjacent OH in the *trans* position reacts more slowly, releasing GDP as the sole UV-active product [146] (Scheme 6B). A nucleophilic attack of the adjacent 2-OH on C1 has been proposed as the mechanism [149]. Interestingly, GDP-mannose behaves differently also in the enzymatic reaction (Section 4.5), even though an intramolecular nucleophile is not involved.

Apart from CDP-neuraminic acid (CDP-Neu5Ac) [150], nucleoside diphosphate sugars react slowly in aqueous solutions. Half-lives of 80 and 40 days have been determined for the cleavage of UDP-Glu and UDP-Gal at pH 7 at 50 °C [146]. Acids enhance the reaction by protonating the phosphate group, and bases increase the nucleophilicity of the attacking OH group by deprotonation [145]. A second-order rate constant of (1.6 ± 0.2)·10^−2^ dm^3^ mol^−1^ s^−1^ at 25 °C has been determined for the base-catalyzed cleavage of UDP-Gal [148]. The temperature dependence of the alkaline cleavage is fairly modest. Rate constants for the cleavage of UDP-Glc in 5 mM NaOH at different temperatures give an activation energy *E*_a_ value of 61.8 kJmol^−1^ [149] and allow a rough estimation of two-fold rate increase for every ten degrees [148].

**Scheme 7:**
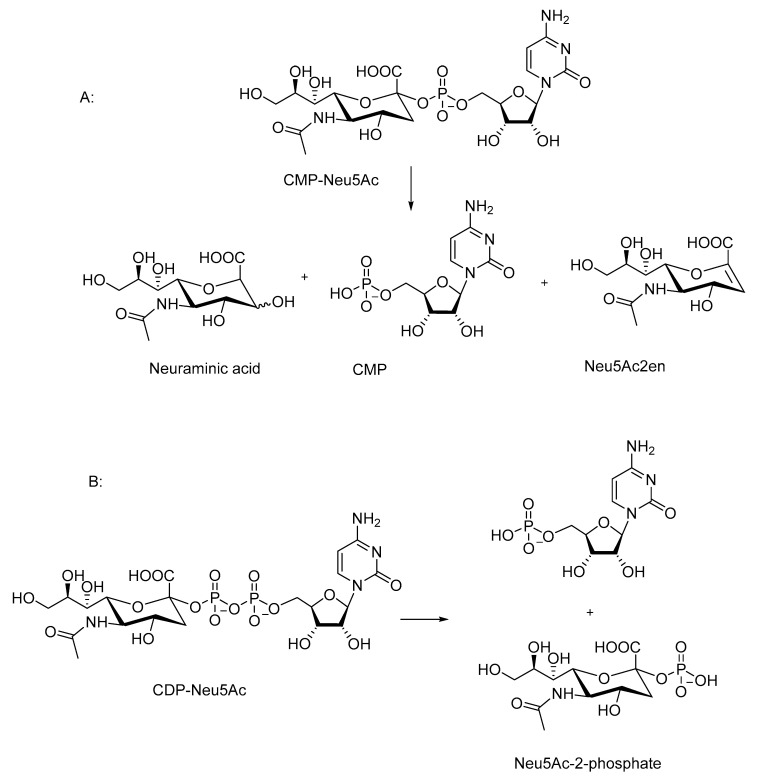
Chemical decomposition of sialic acid containing nucleotide sugars. (**A**): Decomposition of CMP-neuraminic acid (CMP-Neu5Ac) to neuraminic acid, cytidine monophosphate (CMP) and 2-deoxy-2,3-dehydro-*N*-acetylneuraminic (Neu5Ac2en). (**B**): Decomposition of CDP-neuraminic acid (CDP-Neu5Ac) to CMP and neuraminic acid 2-phosphate.

CDP-Neu5Ac [150] and CMP-Neu5Ac [150,151] (Scheme 7) are significantly more reactive than the nucleotide sugars described above. The fastest decomposition is observed under acidic conditions: CDP-Neu5Ac decomposes nearly completely in half an hour at room temperature in 60% acetic acid [150]. CMP-Neu5Ac is only a little less reactive [150,151]. For comparison, the half-lives of UDP-Gal, UDP-Glc, and GDP-Man in 10 mM HCl at 50 °C are 1, 4, and 8 h, respectively [146]. The reactivity of CDP-Neu5Ac decreases only a little as the pH increases: 75% of the starting material is cleaved in an hour at pH 8 at room temperature [150]. The reactivity of CMP-Neu5Ac depends more clearly on pH below pH 7, and a half-life of two days can be estimated at pH 8 and at 25 °C [151], indicating a hundred-fold reactivity difference between the two sialic acid derivatives at pH 8. The rate of the decomposition of CMP-Neu5Ac is practically independent of pH at the pH range 7–11 [151,152].

The numbers presented above give a rough estimation of the reactivity of sialic acid derivatives in comparison to that of other nucleotide sugars. Thus, CMP-Neu5Ac and CDP-Neu5Ac are approximately 250 and 25,000 times as reactive under neutral conditions as UDP-Gal, respectively. The reactivity difference has been attributed to the presence of the carboxylic acid group that enhances both the acid-catalyzed reaction with the oxocarbenium ion intermediate [150], as well as the substitution at the phosphorous [150,152]. The role of the carboxylic acid group has been proven by Kajihira [150] in experiments with carboxylic acid ester and amide derivatives, as well as with ^18^O-labeled substrates.

The decomposition of CMP-Neu5Ac yields 5′-CMP, Neu5Ac, and 2-deoxy-2,3-dehydro-*N*-acetylneuraminic (Neu5Ac2en) as the products (Scheme 7A). [151,152]. The proportion of Neu5Ac2en producing elimination increases at a higher pH. In 30% ammonium hydroxide at 52 °C, the elimination accounts for approximately 40% of the cleavage reaction [152]. The decomposition of CDP-Neu5Ac results in a formation of two monophosphate products (Scheme 7B) [150].

Various metal ion catalysts have been shown to enhance the cleavage of nucleotide sugars [144,146,147]. The catalytic activity of Cu complexes is significant even under neutral conditions. The reaction of UDP-Glc is enhanced in the presence 5 mM CuBiPy at pH 7and 50 °C by a factor 10^4^ and at least a 1000-fold rate enhancement is observed for GDP-Man [146]. Similarly to the reactions of other oligophosphate compounds [153], the reaction of UDP-Gal and UDP-Glc show a second-order dependence on the concentration of CuTerPy, which is possibly due to the dimerization of the catalyst [146]. A similar effect was not observed with GDP-Man. The products of the metal ion-promoted reactions are similar to those formed in alkaline cleavage [144,146,147].

The rate of the metal ion-promoted reactions depends also on pH. While Mn^2+^ promotes the cleavage of UDP-Glc under neutral condition [144], rate enhancement by Mg^2+^ is observed only at a higher pH. At pH 9 and 37 °C, the concentration of UDP-Glc is halved in two hours [147]. Electrophilic catalysis by metal ion catalysts most likely contributes significantly to the catalysis by metal ions and their complexes. However, the fact that the structure of the nucleotide sugar affects the catalytic activity and the concentration dependence suggests that the catalysts may interact also with the nucleophile [146].

## 6. Synthesis of Nucleotide Sugars

### 6.1. Chemical Synthesis

Synthetic methods for nucleotide sugar preparation have been thoroughly reviewed recently [154]. For this reason, the literature covering only the last decade is reviewed here. The synthesis of nucleoside diphosphate analogues has also been discussed in review articles recently [18,155].

**Scheme 8:**
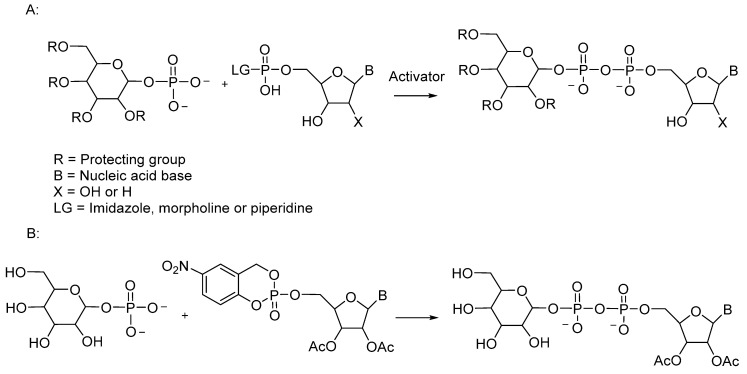
Chemical synthesis of nucleotide sugars. (**A**): Synthesis by coupling two P(V) compounds. (**B**): Synthesis by the *Cyclo*Sal method with a sugar-1-phosphate and a *Cyclo*Sal derivative of a protected nucleoside monophosphate as staring materials.

The pyrophosphate bridge in sugar nucleotides is most often prepared by a coupling reaction between two P(V) species (Scheme 8A). A sugar phosphate is a nucleophile that attacks on the nucleotide derivative with a leaving group. Nucleoside phosphoramidates, such as phosphorimidazolides [156,157], phosphoromorpholidates [158,159,160,161,162], and piperidates [163], have been used in the synthesis. Variable yields have been reported with morpholino derivatives [158], and the formation of UDP-l-rhamnose, for example, has been reported to be five times faster with a phosphoropiperidate as the reagent than with the corresponding phosphoromorpholidate [163].

The synthetic reactions need an acidic activator, and *N*-methyl imidazolium chloride (NMIM) [158], 4,5-dicyanoimidazole (DCI) [162,163], and tetrazole [160], as well as MgCl_2_ [157], have been used in organic solvents. Tetrazole is generally regarded as a less efficient activator than the imidazolium compounds NMIM and DCI. Consistent with this, a comparison of several activators in the preparation of UDP-l-rhamnose, has shown that while the yields obtained are approximately the same, the reaction time with tetrazole is ten times longer than with 3,4-dicyanoimidazole [163]. Even higher yields and faster reactions were obtained with MgCl_2_ activation, but both varied with the structure of the sugar nucleotide [157].

The reactions are usually carried out in an aprotic solvent. However, the choice of an optimal solvent may also depend on the structure of the desired product. The synthesis of GDP-d-mannuronic acid in *N,N*-dimethylformamide (DMF) with DCI as an activator failed to produce any product, but in pyridine, the desired product was obtained in 46% yield in 120 h when the mannuronic moiety was protected [162]. Unprotected mannuronic acid 1-phosphate did not react at all in either of the solvents.

A less extensively used strategy is to use *Cyclo*Sal-protected nucleotides and sugar 1-phosphates as reagents in an organic solvent (Scheme 8B). A number of different types of nucleotide sugars have been synthesized in yields ranging from moderate to excellent [164]. The method can be used also for the synthesis of CMP-sialic acids [165]. In this case, the nucleophile is the axial 2-OH that is deprotonated to an oxyanion by NaH under the reaction conditions. Various *N*-acyl derivatives of CMP-neuraminic acid have been prepared in 50–70% yield. CMP-activated neuraminic acid derivatives have been synthesized also by using phosphoramidite chemistry [166]. Fluorescently labeled derivatives have been prepared by coupling a nucleoside phosphoramidite building block and a protected sialic acid derivative with tetrazole as an activator [166].

### 6.2. Enzymatic Methods

Enzymatic and chemoenzymatic processes involving nucleotide sugars as products or as glycosylating agents are often coupled, and they are difficult to discuss totally separately. Hence, discussion about glycosylation can not be totally avoided, although there is an emphasis in this section on the synthesis of nucleotide sugars. The following discussion aims at giving a brief overview on the methodology and a few specific examples of the reactions and their applications.

Nucleotide sugars can be synthesized by one-pot syntheses utilizing enzyme cascades to produce nucleotide sugars from easily obtainable and affordable starting materials. A nucleotide sugar-producing cascade can be combined with a glycosylation reaction, and often, the nucleotide released is recycled to regenerate the nucleotide sugar. Processes of several steps with multiple enzymes are referred to as OPME reactions (One Pot, Multiple Enzymes), and single OPME reactions can be treated as modules to build more complex systems, as will be discussed in the following section.

The processes outlined above can be fully enzymatic or chemoenzymatic, combining chemical and enzymatic steps. Unnatural nucleotide sugars are often prepared by chemoenzymatic synthesis with chemical steps to produce the sugar or sugar phosphate and with enzymatic steps for the synthesis of the nucleotide sugar. Enzymatic reactions can be carried out in vitro using natural or recombinant enzymes, but also in whole cells expressing desired enzymes. Enzymatic or chemoenzymatic oligosaccharide synthesis using nucleotide sugars as donors can be done also on solid phase, and automated methods are being developed. These vast topics, chemoenzymatic methods for the synthesis of nucleotide sugars [167,168], oligosaccharides [169,170], or glycocojugates [171,172], glycoenzyme engineering [173,174,175], and solid-phase and/or automated methods [176] fall outside the scope of this review and are not discussed here.

#### 6.2.1. Enzymatic Synthesis of Nucleotide Sugars

The first step of an enzymatic synthesis of a nucleotide sugar is often a kinase-catalyzed phosphorylation of a sugar with a nucleoside triphosphate as the phosphoryl donor. In a one-pot reaction, kinase first phosphorylates the sugar, producing a sugar 1-phosphate, and a pyrophosphate-forming enzyme completes the synthesis. For example, UDP-GlcNAc has been synthesized via a kinase-dependent pathway using a pyrophosphorylase [177] or a uridylyl 1-phosphate transferase to produce the pyrophosphate bridge [178]. A synthesis using UMP as starting material has also been reported [179].

Kinase-dependent pathways have been reported for the synthesis of various nucleotide sugars including UDP-Gal and UDP-GalNAc [177], GDP-mannose [180], GDP-fucose [181], as well as nucleotide sugars with unnatural sugar moieties [167,182,183,184]. GDP-mannose has been synthesized also via a kinase-independent route from mannose 1-phosphate produced in two steps from sucrose [185].

UDP-glucose can be synthesized in a single step from sucrose and UDP by a sucrose synthase [178,186,187,188]. The use of UDP can be avoided by a two-step process with UMP as a starting material [189]. A whole-cell cascade with starch as a starting material has also been reported [190]: A glucan phosphorylase produces glucose 1-phosphate that is converted to UDP-glucose by a pyrophosphorylase. UDP-glucose produced can be further converted to UDP-glucuronic acid (UDP-GlcA) by oxidizing the hydroxymethyl group with an NADH-dependent dehydrogenase [178,186,190]. The NADH coenzyme can be recycled by an oxidase [178,186,190]. Another route to UDP-GlcA uses glucoronic acid as the starting material, thus avoiding the NADP-dependent oxidation step [191]. An oxidation of UDP-Glc to UDP-GlcA followed by decarboxylation by UDP-xylose synthetase has been used to produce UDP-xylose [192]. UDP-xylose has been prepared also in a single enzymatic step from chemically synthesized sugar phosphate [193].

Sialic acid nucleotide sugars have been produced with ManNAc and puryvate as starting materials. An aldolase catalyzes a formation of a sialic acid, which in the presence of CTP is converted to corresponding nucleotide sugar by CMP–sialic acid synthetase [181,183].

#### 6.2.2. Enzymatic Synthesis Coupled with Glycosylation

Individual cascades can be used as modules to build more complex systems [178,194,195]. As an example, two different three-module one-pot syntheses for production of hyaluronan have been reported [178,196]. In a system reported by Eisele et al. [178], one module produces UDP-GlcA from sucrose and UDP, and another synthesizes UDP-GlcNAc by a kinase-dependent pathway. The products are polymerized in the third module by a hyaluronan synthase with a release of UDP. UDP is used by sucrose synthase for UDP-Glc synthesis and by a pyruvate kinase to produce UTP for the synthesis of UDP-GlcNAc. In the system developed by Li et al. [196], glucuronic acid is used as a starting material in a kinase-dependent pathway to produce UDP-GlcA.

Nucleotide sugars are expensive starting materials, which restricts their use in large-scale synthesis. However, a combination of nucleotide sugar-producing cascades with a recycling of nucleotides and NADH has enabled a gram-scale synthesis of nucleotide sugars and glycosylation products. The development and optimization of synthetic procedures requires careful analysis of the reaction parameters, and efficient analysis methods are essential in such a work. For example, NMR spectroscopic methods [197,198] and multiplexed capillary electrophoresis [199] have been used.

With optimized reaction systems, multigram production of UDP-Gal, UDP-GlcNAC, UDP-GalNAc [177], and UDP-Xyl [192] has been achieved. An up-scaled synthesis produced 23.3 g UDP-GalNAc with a space-time yield of 19.4 g dm^−3^ h^−1^ [177]. A UDP-xylose to prepare azido derivatized Lewis antigen structures has also been reported [183]. The rapidity of the production is another advantage of optimized OPME reactions. As an example, a hyaluronan polymer of M_w_ of 2.3 MDa has been prepared in a laboratory scale in 10 h [175]. Similar efficiencies have been reported also in the glycosylation of a chemically synthesized disaccharide with UDP-GlcA [183] and in the biosynthesis of oligosaccharides raffinose and stachyose with sucrose and UDP as starting materials [200].

## 7. Conclusions

The formation and reactions of nucleotide sugars both under biological and chemical conditions have been extensively studied. Information on the biosynthetic routes and biological roles, and on the structures of molecules involved in these processes, is essential in identifying targets for therapeutic methods. It is of particular importance to identify the differences between human and bacterial structures and processes.

Understanding the catalytic mechanisms of nucleotide sugar-utilizing enzymes is still increasing, and new enzyme superfamilies have been recently discovered. The different reactivity under chemical conditions underlines the importance of the stabilization of the oxocarbenium ion-like transition state in glycosylation reactions. Studies on the chemical reactivity show also that a change in the structure or reaction conditions may have a significant effect on the reactivity of nucleotide sugars.

Methods of chemical synthesis have been developed and natural and modified nucleotide sugars have been synthesized. Protecting groups are usually needed in the synthesis, and the choice of optimal reaction conditions depends on the structure of the target molecule. Nevertheless, chemical synthesis provides the means for the production of unnatural nucleotide sugars needed in the synthesis of modified oligosaccharides, as potential enzyme inhibitors, and in diagnostic methods.

The development of chemoenzymatic methods for the synthesis of nucleotide sugar and oligosaccharides is an example of the power of cooperation between scientists of different fields. Methods that enable a one-pot synthesis of modified oligosaccharides in gram-scale yields provide materials for the purposes of future research and drug development.
